# Management of hypocalcemia following thyroid surgery in children

**DOI:** 10.3389/fped.2023.1151537

**Published:** 2023-05-04

**Authors:** Andrea Romera, Lourdes Barragán, Lucía Álvarez-Baena, Erika Sanjuán, Javier Ordóñez, María Antonia García-Casillas, Marina Mora, María Sanz

**Affiliations:** ^1^ Department of Anesthesiology, Pediatric Section, Gregorio Marañón University Hospital, Madrid, Spain; ^2^ Department of Pediatric Surgery, Gregorio Marañón University Hospital, Madrid, Spain; ^3^ Department of Pediatric Endocrinology, Gregorio Marañón University Hospital, Madrid, Spain

**Keywords:** hypocalcemia, pediatric, children, thyroidectomy, protocol

## Abstract

**Introduction:**

Pediatric post-thyroidectomy hypocalcemia management varies significantly from hospital to hospital. The current study has two aims: first, we evaluate demographic data in all pediatric patients submitted to thyroid surgery in our Spanish tertiary hospital over 20 years; secondly, we describe the way that hypocalcemia was diagnosed and treated in that period and present a multidisciplinary protocol for perioperative management of this condition.

**Methods:**

This is a retrospective and observational study of all patients from 0 to 16 years old who underwent thyroid surgery from 2000 to 2020 at our institution. Demographic, surgical and electrolyte data were recorded from the electronic database.

**Results:**

From 2000 to 2016, pediatric thyroid surgery at our institution was performed on 33 patients without a consistent approach or standard electrolyte management. A protocol for perioperative management of these patients was introduced in 2017, and applied to 13 patients. In 2019, the protocol was assessed and updated following a case of symptomatic hypocalcemia. From 2000 to 2016, 47 pediatric patients in all underwent thyroid surgery. We registered eight asymptomatic hypocalcemias. One child developed symptomatic hypocalcemia. Two patients have permanent hypoparathyroidism.

**Discussion:**

Our incidence of general complications following thyroidectomy was low; hypocalcemia was the most prevalent. All the cases of hypocalcemia submitted to the protocol were identified early by iPTH measurements. Intraoperative iPTH levels and percentage drop from baseline could help stratify patients according to their risk of hypocalcemia. High risk patients require immediate postoperative supplementation, including calcitriol and calcium carbonate.

## 1. Introduction

There is an extensive literature on adult thyroidectomy but relatively few reports on thyroidectomy in pediatric patients. There are known and important differences between these two populations, such as a higher incidence of postoperative complications in pediatric patients (1–4). Hypocalcemia, the most frequent complication, is caused by the removal of the parathyroid glands or a compromise to their vasculature (5, 6). It prolongs hospital stays, increases monitoring, and can lead to high morbidity (7, 8). Pediatric post-thyroidectomy hypocalcemia rates vary widely in the literature (9–11). It is difficult to foresee which patients are likely to develop it, because its symptoms usually manifest 24–48 h after surgery (12), when some patients have been already discharged from the hospital.

Several reports in adults have demonstrated the utility of measuring the level of postoperative intact parathyroid hormone (iPTH) to help predict which patients will develop hypocalcemia following total thyroidectomy (8). Given the short half-life of PTH, the decline is expected to be rapid, well before the decrease in serum calcium (3). Freire et al. evaluated the accuracy of intraoperative iPTH to identify postoperative hypocalcemia early in children (10) and Jiang et al. demonstrated the utility of iPTH in predicting and managing postoperative hypocalcemia following thyroidectomy in pediatrics (4).

Many strategies have been designed to minimize hypocalcemia following thyroid surgery, such as multiple biochemistry measurements or prophylactic administration of calcium and vitamin D. Electrolyte management also varies significantly from hospital to hospital (8), leading several international articles to highlight the need for more detailed retrospective institutional reviews. The adoption of a standard approach to predict, identify, and treat this condition would help reduce morbidity (13). In general, literature describing calcium management in the pediatric population is rare (8) and the need for postoperative calcium replacement algorithms for children has also been shown by many authors, together with the need for closer monitoring and routine postoperative intensive care unit admission (2). In 2017, a post-anesthesia care unit (PACU) for prolonged-stay pediatric patients was created at our institution. A clinical protocol for hypocalcemia detection and electrolyte management was also developed.

The current study has two aims: First, we evaluate demographic data in all pediatric patients submitted to thyroid surgery in our tertiary hospital over 20 years. Secondly, we describe the way that hypocalcemia was diagnosed and treated in that period and present a multidisciplinary protocol for perioperative management of this condition.

## 2. Materials and methods

### 2.1. Study design

This is a retrospective and observational study of all patients from 0 to 16 years old who underwent thyroid surgery from 2000 to 2020 at the Pediatric Section of Gregorio Marañón University Hospital in Madrid (Spain). In Spain, patients older than 16 are being managed by the adult surgeons and endocrinologists.

Demographic and surgical data were recorded. Variables collected included patient characteristics [age, sex, American Society of Anesthesiologists’ Physical Status Classification (ASA)], type of thyroid disease, type of intervention, and length of hospital stay. Regarding electrolyte management, all preoperative, intraoperative, and postoperative measurements of corrected total serum calcium (CT-Ca^2+^), phosphorus, magnesium, and iPTH levels were collected in each patient. The following complications were registered: recurrent laryngeal nerve injury, infection, hematoma, fistula, Horner's syndrome, and hypocalcemia. Hypocalcemia was defined as any CT-Ca^2+^ of <8 mg/dl. Hyperphosphoremia was defined as any phosphorus >7 mg/ml. Hypomagnesemia was defined as any magnesium <1.8 mg/dl. iPTH levels should not be elevated with 25-hydroxy vitamin D above 20 ng/ml.

Long-term hypocalcemia was defined as necessitating supplementation at around six months after surgery. Data were collected from the hospital records database (Health Care Information System®) and recorded on an Excel® (Microsoft Corporation, United States) spreadsheet.

### 2.2. Periods

- Non-protocolized period: From 2000 to 2016, the pediatric thyroid surgery had no consistent approach or standard electrolyte management at our institution. All patients spent two hours in the PACU, and the rest of their hospital stay was in their room under surgical supervision. Serum concentrations of calcium, phosphorus, and magnesium were checked both pre- and postoperatively according to the doctor's personal criteria. iPTH (chemiluminescence microparticle immunoassay, Alinity; normal range: 15–68 pg/ml) was assessed at least once either in the PACU or after discharge as part of the endocrinology follow-up. No intraoperative iPTH measurements were conducted. The decision to start the maintenance solution (described below) with calcium gluconate as well as the oral treatment with calcium carbonate and 1,25-(OH)2-vitamin D (calcitriol) was based exclusively on the type of surgery. That way, all cases of total thyroidectomy were treated regardless of their electrolyte levels. Patients who underwent hemithyroidectomy did not receive preventive supplements of any kind.
- Original protocol period: A protocol for perioperative management of children submitted to thyroid surgery was introduced in 2017. It was based on published evidence and expert opinion. Pathway teams included representatives from pediatric endocrinology, pediatric surgery and pediatric anesthesiology. The routine admission in the PACU for 24 hours was compulsory for all patients. The protocol consisted of preoperative determinations of iPTH, CT-Ca^2+^, phosphorus, magnesium, thyroglobulin, thyroglobulin antibodies, albumin, creatinine, calcitonin, free T4, TSH, and 25-hydroxyvitamin D (calcidiol). We also extracted a triple iPTH determination before the surgical incision (iPTH-B) as well as 5 (iPTH-5) and 10 (iPTH-10) minutes after thyroid removal. A peripherally inserted central venous catheter was placed in the operating room to avoid repeated puncture. Patients in both the operating theater and in the PACU were treated with a maintenance solution of 500 cc of plasmalyte 148, 5 cc ClK 2M, and 10 cc of 10% calcium gluconate. This solution, containing 0.18 mg of elemental calcium per cc, was set at two-thirds of the basal needs, according to the Holliday and Segar method (14). This perfusion was settled during at least the first 12 hours and until oral feeding was restored. A single dose of 30 mg/kg of intravenous cefazolin and of 0.1 mg/kg of intravenous ondansetron was given during surgery. An attempt to look for all the parathyroid glands and preserve their blood supply was always made during the operation. Clinical features associated with hypocalcemia were closely monitored in the PACU. Biochemical confirmation was checked immediately if there were any concerning examination findings. Magnesium and phosphorus values were rechecked if there were persistent biochemical hypocalcemia. Oral levothyroxine was started the morning after the surgery. Discharge criteria were CT-Ca^2+^ > 8 mg/dl in the two last determinations, optimal pain control, no fever, and no signs of bleeding.

Oral calcium was started in the case of hypocalcemia and/or iPTH-5 and/or iPTH-10 lower than 80% of basal value. Such treatment used 50 mg/kg/day calcium carbonate, which contains 40% elemental calcium. Intravenous calcium gluconate was also given in the case of CT-Ca^2+^ levels lower than 8 mg/dl, at doses of 1.5 mEq of calcium/kg/day. The decision to add calcitriol to the treatment was based on the theoretical level of parathyroidectomy predicted by the surgeon and registered hypocalcemia. Calcitriol was started 72 h after surgery in cases of partial parathyroidectomy and CT-Ca^2+^ < 8 mg/dl. Confirmed total parathyroidectomy led to calcitriol started 24 hours after surgery.

### 2.3. Ethics approval

This was an observational study, and permission from patients and approval from the ethics committee were not required.

## 3. Results

### 3.1. Demographic and surgical data

From 2000 to 2020, 47 pediatric patients underwent thyroid surgery; 29 of them were female. The median age was 9 years (range 3–15 years). All patients were healthy ASA I or II. The median follow-up was 29 months (range 6–171 months). The median hospital stay was 27 hours (range 24–45 h).

There were 29 cases of Multiple Endocrine Neoplasia Type 2 (MEN 2) A, one case of MEN 2B, five papillary carcinomas, five follicular adenomas, four multinodular goiters, one follicular carcinoma, one thyroglossal duct papillary carcinoma, and one Graves-Basedow syndrome. Regarding MEN 2 syndrome surgeries, 28 patients had prophylactic thyroidectomies. The pathological study of the other two patients (both with MEN2A) revealed a medullary thyroid carcinoma. Total thyroidectomy was performed in 41 patients. Four follicular adenomas and one multinodular goiter underwent hemithyroidectomy. None of these developed intraoperative complications. One case presented with recurrent laryngeal nerve lesion and mild dysphonia right after surgery, but it disappeared within hours. All surgeries were performed by the same surgical team.

### 3.2. Electrolyte and iPTH data

Over the non-protocolized period, 33 patients underwent thyroid surgery. Six cases of total thyroidectomy presented asymptomatic hypocalcemia. They had normal renal function and normal magnesium levels. All were treated and discharged with calcium carbonate. The replacement therapy was discontinued in the following six months in five cases. One case from 2006 still has permanent hypoparathyroidism.

In 2017, the aforementioned protocol was developed and 13 patients were submitted to it (Table 1). None of them developed intraoperative complications. Postoperative complications included one case of mild dysphonia. In this group, we registered one asymptomatic hypocalcemia (6th patient, as in Table 1) in a patient with preoperative calcidiol deficiency and elevated baseline iPTH. It is noticeable that iPTH-5 dropped by 50% and iPTH-10 by 40% with respect to the baseline. First and second postoperative iPTH determinations were also pathological, with a low of 18% on arrival to PACU. The child was treated and discharged with oral calcium, calcitriol and normal calcium levels. Calcium replacement therapy was discontinued after three months.

**Table 1:** Characteristic and electrolyte data of the patients submitted to the original protocol.

	1	2	3	4	5	6	7	8	9	10	11	12	13
Diagnosis	MEN 2A	PC	MEN 2A	MEN 2B	MEN 2A	MG	MEN 2A	MEN 2A	MEN 2A	MEN 2A	MG	MEN 2A	MEN 2A
Age (years)	5	12	3	11	6	12	6	4	4	5	11	5	11
Sex	Female	Male	Female	Male	Female	Female	Male	Male	Female	Male	Female	Male	Female
iPHT-B	54	73	65	62	60	214	36	75	61	68	81	90	86
iPHT-5	28	71	62	54	65	106	24	72	68	64	76	76	5
iPHT-10	36	90	63	64	62	127	35	74	49	52	70	72	<3
iPTH po-1	6	22	25	20	31	39	21	65	3	25	35	52	<3
iPTH po-2	49	61	55	57	52	67	30	69	50	59	70	72	<3
CT-Ca^2+^ pre	9.9	9.8	9.2	9.8	9.6	9.8	9.7	11.1	9.9	9.5	9.9	9.8	7.3
CT-Ca^2+^ po-1	10.2	9.9	9.2	10	9.7	7.5	9.8	10.3	9.2	9.1	9.1	9.3	7.1
CT-Ca^2+^ po-2	9.9	9.3	10.3	10	9.3	7.6	9.2	9	9.7	9	9.2	8.5	7.4
CT-Ca^2+^ po-3						8.1						8.8	8
Mg^2^ pre	2.3	1.8	2.7	2.1	2.9	2.2	1.9	2.1	2.1	2.3	1.9	2.6	2.2
Mg^2^ po-1	2.1	2	2.1	2.3	2.6	2.2	2.2	2.4	2.3	2.1	2	2.1	2.5
P pre	6.2	5.2	5.9	5.4	4.4	4.9	5	5.3	5.9	5.5	4.7	5.4	5.2
P po-1	5.1	4.9	5.9	5.6	4.9	5.9	5.5	4	4	5	5.1	6.1	7.4
P po-2	6.6	5.7	4.3	5.3	4.9	4.1	5.9	5.1	5.3	5.2	4	6.7	8.4
25-OH-VitD	35	29	30	22	31	16	33	28	30	33	27	18	16
Others			Dysphonia			AH TD							SH TD

Pathological results are in grey. PC, papillary carcinoma; MG, multinodular goiter; F, female; M, male; iPHT-B, basal intact PTH, in pg/ml; iPHT-5, intact PTH 5 min after the thyroid's removal, in pg/ml; iPHT-10, intact PTH 10 min after the thyroid's removal, in pg/ml; CT-Ca^2+^, corrected total calcium, in mg/dl; Mg^2+^, magnesium, in mg/dl; 25-OH-VitD, preoperative calcidiol, in ng/ml; Pre, preoperative; Po-1, determination on arrival to PACU; Po-2, determination 6 h after Po-1; Po-3, last determination before hospital discharge; AH, asymptomatic hypocalcemia; SH, symptomatic hypocalcemia; TD, discharge with treatment for hypocalcemia.

In 2019, our 13th patient developed symptomatic hypocalcemia, despite the implementation of the protocol, and remains with permanent hypoparathyroidism. This was an 11-year-old girl who underwent a total thyroidectomy due to MEN2A. She had calcidiol deficiency. PTH-5 was less than 80% of basal value and the PTH-10 was undetectable. Calcium infusion was settled according to protocol. Treatment with oral calcium carbonate was initiated 24 hours after surgery (and no calcitriol was given). Patient was discharged 72 hours after surgery, with normal calcium levels. 48 hours after discharge she was readmitted with muscle cramps and spasms, facial twitching and prolonged QTc. All calcium measures were low. Magnesium was normal. Intravenous gluconate calcium 10% was started and she could be discharged 6 hours after, with oral calcium carbonate and calcitriol.

**Figure 1:**
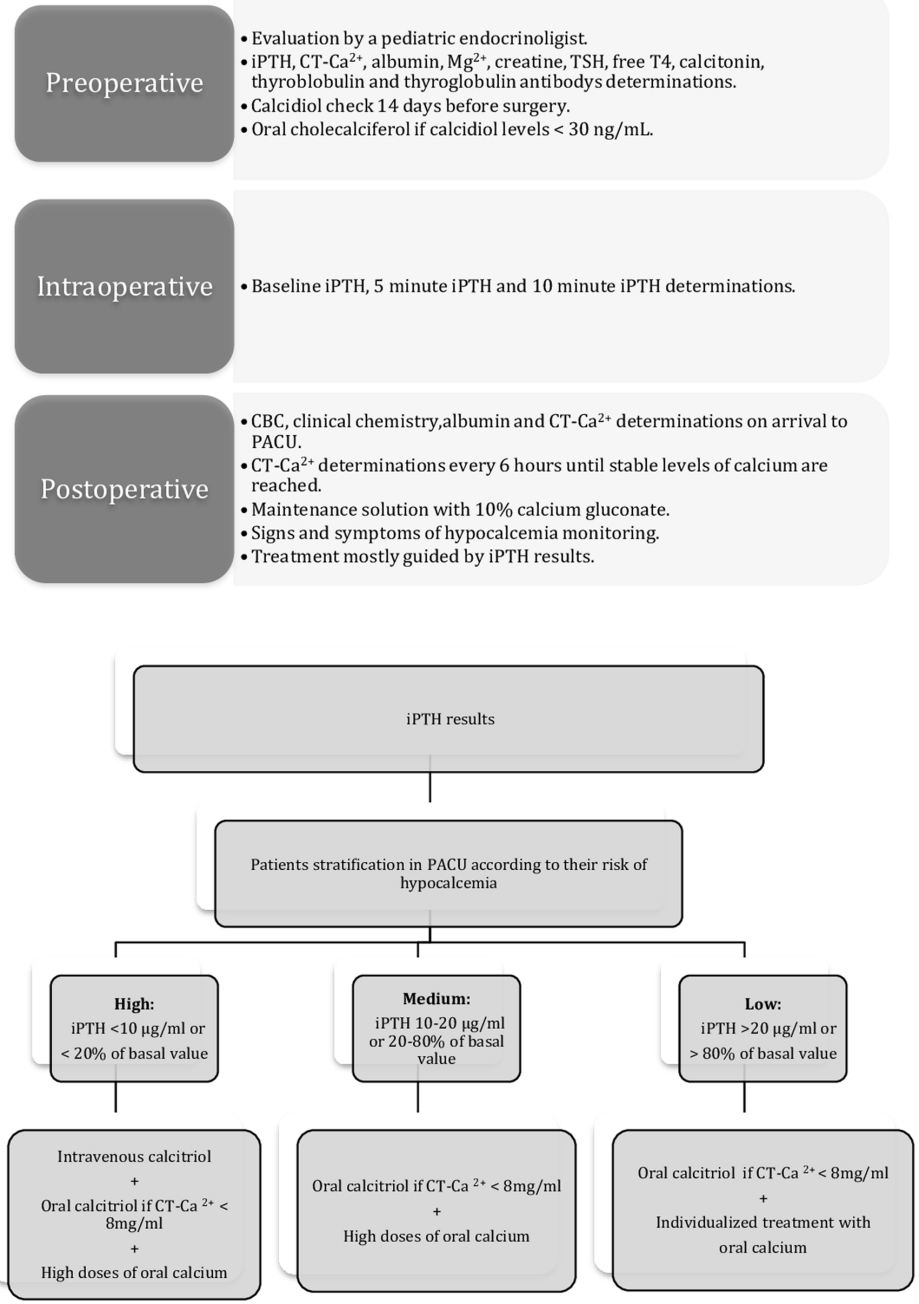
Updated protocol. iPHT, intact PTH; CT-Ca^2+^, corrected total calcium; Mg^2+^, magnesium; iPHT-B, baseline iPTH; iPHT-5, iPTH 5 min after the thyroid's removal; iPHT-10, iPTH 10 min after the thyroid's removal; CBC, complete blood count; PACU, post-anesthesia care unit.

**Figure 2:**
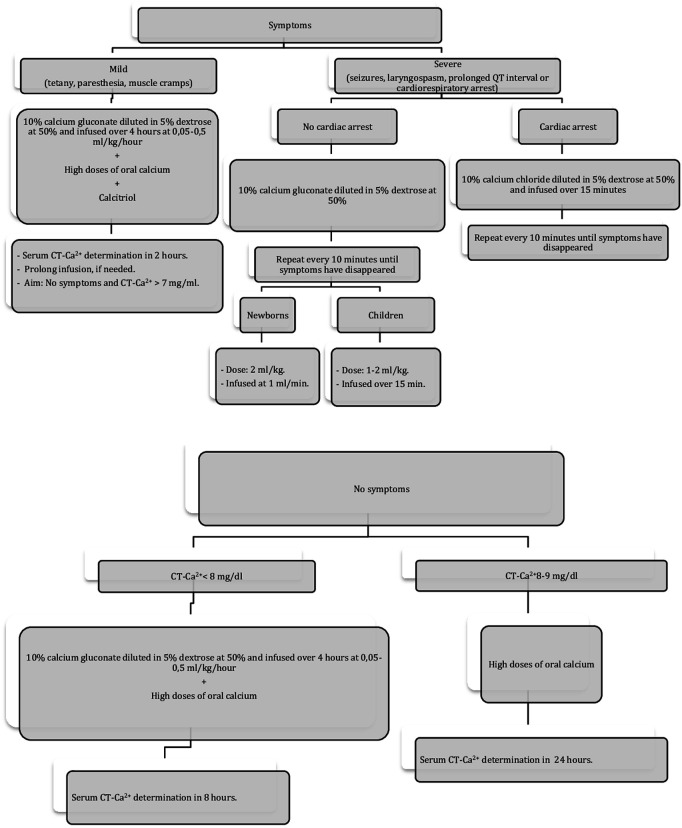
Hypocalcemia treatment. CT-Ca^2+^, corrected total calcium; Hypocalcemia is defined as CT-Ca^2+^ < 8 mg/dl. Each mililitre of 10% calcium gluconate contains 9.3 mg of elemental calcium.

The pathway was then assessed and updated following the case above; it is now focused on the identification of patients at risk of hypocalcemia and on aggressively treating this condition (15, 16) (Figure 1). The modified features are in Table 2:

- To optimize vitamin D levels, calcidiol is checked two weeks before surgery. If calcidiol is <30 ng/ml, then vitamin D3 (cholecalciferol) is prescribed (10) and maintained until the procedure (Table 3).
- Intraoperative iPTH results allow patient stratification according to their risk of hypocalcemia (8, 10): high (iPTH < 10 pg/ml or <20% basal value), medium (iPTH 10–20 pg/ml or 20%–80% of basal value), or low (iPTH > 20 pg/ml or >80% of basal value) (Table 3).
- Higher risk patients require immediate postoperative supplementation including calcitriol and calcium carbonate (Table 3).
- An independent algorithm for the management of hypocalcemia was created (17) (Figure 2).
- If a patient is being discharged on any calcium supplement, then they should be followed up three days after discharge.

**Table 2:** Summary of the main features of the original and the updated protocol.

	Original protocol	Updated protocol
Preoperative calcidiol	Measured	Measured and optimized with cholecalciferol
Oral calcium dose	50 mg/kg/day	Up to 150 mg/kg/day
Postoperative calcitriol	Decision based on identification of the parathyroid glands	Given to high and medium risk patients
Risk stratification	Based on iPTH and CT-Ca^2+^ levels	Based on iPTH levels or percentage drop

**Table 3:** Indications and dosage of calcium and vitamin D supplementations.

Preoperative oral cholecalciferol
- Start two weeks before surgery. - Aim: getting levels of preoperative calcidiol >30 ng/ml. - Oral cholecalciferol dose: - If calcidiol 20–30 ng/ml: • <1 year: 800 units/day. • 1–12 years: 1,000 units/day. • 12 years: 2,000 units/day. - If calcidiol <20 ng/ml: • <1 year: 1,000 units/day. • 1–12 years: 2,000 units/day. • 12 years: 3,000–5,000 units/day.
Postoperative oral calcium (given every 6 h)
- Given as calcium carbonate (contains 40% of elemental calcium). - High risk: 50–150 mg/kg/day of elemental calcium. - Medium risk: 20–40 mg/kg/day of elemental calcium. - Low risk: No treatment or 10–20 mg/kg/day of elemental calcium.
Postoperative calcitriol (given every 12 h)
- High risk (intravenous administration): - <5 years: 0.25 µg/day. - 5–10 years: 0.5 µg/day. - >10 years: 1–2 µg/day. - Medium and low risk (oral administration): - <1 year: 0.04–0.08 µg/day. - 1–5 years: 0.25–0.75 µg/day. - >6 years: 1–2 µg/day.

Over the studied period, only one patient was submitted to the updated pathway.

## 4. Discussion

Our incidence of general complications following total or partial thyroidectomy was low; hypocalcemia was the most prevalent. We had two (4%) patients with permanent hypoparathyroidism and seven (14%) with temporary hypoparathyroidism. Our rates are lower than other figures reported in children (2, 16, 18, 19). In addition, some authors suggest that patients under the age of one (2) or even seven (20) have a higher risk of complications vs. older pediatric patients. While we included all patients under 16 years of age, our median age was nine years, which may have increased the complexity of the cases.

While some authors have shown that high-volume centers and teaching hospitals tend to have better outcomes and less hypoparathyroidism (2), others do not identify any clear association between hospital thyroidectomy surgical volume and complication rate (3) Although the number of annual procedures that constitutes “high-volume” is not clear, specialized pediatric institutions whose surgeons perform 30 thyroidectomies per year are rare in Spain (20). In any case, as Jiang highlights, favorable outcomes can be achieved by a multidisciplinary team of pediatric providers – surgeons, endocrinologists and anesthesists - despite low surgical volume (3). It is clear, though, that pediatric thyroid surgery is safe when performed by specialized personnel (21). Risk surgical factors for hypoparathyroidism following total thyroidectomy include thyroidectomy for definitive treatment of Graves' disease and thyroid cancer (3, 16, 19). The latter is well represented in our cohort, as our tertiary hospital receives complex patients from other healthcare facilities, which makes our figures of malignant pathologies higher. On the other hand, Graves' disease is typical of young adults, and our pediatric section, following Spanish regulations, only treats patients up to 16, which may explain why our cohort includes one patient. In addition, since the risk of thyroid cancer tends to increase with age, our figures may be lower than those of series with older adolescents (3).

In our study, all the cases of hypocalcemia submitted to the protocol were identified early by iPTH measurements. On one side, intraoperative iPTH checks could efficiently predict post-thyroidectomy hypocalcemia in our population and make immediate decisions regarding calcium and vitamin D supplementation. Future studies should be performed in a larger pediatric cohort to determine if they can also predict the timing for parathyroid gland recovery. On the other side, there have not been many publications validating iPTH cutoffs in the pediatric population. Freire (10) demonstrated that PTH assay ≤14 pg/ml 60 min postoperatively had a sensitivity of 90% and specificity of 100% in predicting hypocalcemia. Jiang's iPHT levels (4), taken at least 20 min after surgery, appear to have slightly lower sensitivity with the same high specificity, using a higher PTH threshold of 26 pg/ml.

These diverse iPTH cutoffs may be due to difference in the intact PTH assay used. iPTH normal values depend directly on the laboratory techniques, and it was essential for our institution to define our own reference values. In addition, the relatively young age of our patients, compared to other series, may have resulted in lower iPTH cutoffs than Jiang's. As they highlight, significant increase in baseline PTH levels takes place during puberty and bone growth spurts. Jiang hypothesize that, in this population, that is not ours, a higher serum PTH level may be required during the postoperative period to maintain normal calcium homeostasis.

Elevated baseline iPTH levels can be explained due to the vitamin D deficiency. That was the case of patient 6, who had an even higher iPTH than the low-risk group, and higher than Jiang's cutoff of 26, but it was correctly classified as moderate-risk based on the 50% drop of iPTH-5. Using both the numeric cutoffs and the percentage of drop with respect to baseline is an approach worth studying further, although it raises the costs.

Our original pathway was focused on detecting biochemical and symptomatic hypocalcemia in the immediate postoperative period when transient hypoparathyroidism is most common. This aim was accurately achieved. All patients had a similar length of stay, which means that the management of children at risk of hypocalcemia while in the PACU was also correct. However, it is clear that we needed to update the protocol to prevent severe and symptomatic hypocalcemia, and to treat hypoparathyroidism more expediently. Linked to this, the study of preoperative levels of vitamin D and its aggressive repletion should be emphasized. Of note, prolonged treatments ahead of the surgery, longer than 14 days, may be needed for the levels to rise.

One concern for hypocalcemia can lead to prolonged hospitalization of patients who would otherwise be discharged early after the operation or even be outgoing patients in large academic centers (20). Routine oral calcium supplementation has been proposed empirically (22) or even given per protocol to promote outpatient pediatric surgery, but that would involve unnecessary treatment of many patients (20). It is a fact that replacement strategies using PTH have allowed adult thyroid surgeries to be performed as outpatient. However, in pediatric patients, the decline in serum calcium may be significantly delayed, resulting in many authors stating that pediatric patients following thyroidectomy at risk for hypocalcemia should be monitored on an inpatient basis (3), such a PACU.

The decision to treat hypocalcemia is based on the severity of symptoms. If intravenous infusions are contemplated, then hospitalization in an intensive care unit with access to cardiac monitoring and rapid ionized calcium determinations is ideal for optimal management and safety. In general, pediatric electrolyte management is a major challenge in the PACU for different reasons. First, most guideline recommendations are based on expert opinion. Second, most anesthesiologists are not familiar with calcium and vitamin D management and often require advice from the endocrinologist. In addition, symptoms may not be self-reported, and phlebotomy may be more challenging in a child (8). Standardization of post-thyroidectomy care for all patients in our PACU provided insight into our clinical practice and has been useful in identifying areas that could be improved. We note that the protocol was enthusiastically embraced by all care providers, and they gained experience managing endocrine-specific electrolyte complications. Furthermore, hypocalcemia can delay discharge (13), which explains why pediatric thyroidectomies can benefit from short standardized admissions in specialized PACUs regardless of their lab values. Further studies are needed to determine whether these pediatric units enhance early hospital discharge or not.

The limitations of this study include the retrospective study design and the small number of patients to whom the initial protocol was applied (*n* = 13), and having only one patient in the updated version that is now under evaluation. This cohort had a relatively small number of patients with cancer and Graves' disease, that may be more likely to develop hypocalcemia. Although our institution is a teaching hospital with a pediatric division, we acknowledge that the number of patients in our study is relatively small. However, analysis of the data led to adjustments in the dosage of calcium and vitamin D supplementation; this reconfirmed our original decision to reduce routine calcium and electrolyte determinations. Of note, few pediatric thyroidectomies have been performed since then due to the coronavirus pandemic. Further studies are needed to assess the real impact of these modifications.

## Data Availability

The raw data supporting the conclusions of this article will be made available by the authors, without undue reservation.
